# Gut Dysbiosis in Animals Due to Environmental Chemical Exposures

**DOI:** 10.3389/fcimb.2017.00396

**Published:** 2017-09-08

**Authors:** Cheryl S. Rosenfeld

**Affiliations:** ^1^Bond Life Sciences Center, University of Missouri Columbia, MO, United States; ^2^Biomedical Sciences, University of Missouri Columbia, MO, United States; ^3^Thompson Center for Autism and Neurobehavioral Disorders, University of Missouri Columbia, MO, United States; ^4^Genetics Area Program, University of Missouri Columbia, MO, United States

**Keywords:** endocrine disrupting chemicals, arsenic, nanoparticles, lead, heavy metals, air pollution, gastrointestinal system, gut-microbiome-brain axis

## Abstract

The gut microbiome consists of over 10^3^–10^4^ microorganism inhabitants that together possess 150 times more genes that the human genome and thus should be considered an “organ” in of itself. Such communities of bacteria are in dynamic flux and susceptible to changes in host environment and body condition. In turn, gut microbiome disturbances can affect health status of the host. Gut dysbiosis might result in obesity, diabetes, gastrointestinal, immunological, and neurobehavioral disorders. Such host diseases can originate due to shifts in microbiota favoring more pathogenic species that produce various virulence factors, such as lipopolysaccharide. Bacterial virulence factors and metabolites may be transmitted to distal target sites, including the brain. Other potential mechanisms by which gut dysbiosis can affect the host include bacterial-produced metabolites, production of hormones and factors that mimic those produced by the host, and epimutations. All animals, including humans, are exposed daily to various environmental chemicals that can influence the gut microbiome. Exposure to such chemicals might lead to downstream systemic effects that occur secondary to gut microbiome disturbances. Increasing reports have shown that environmental chemical exposures can target both host and the resident gut microbiome. In this review, we will first consider the current knowledge of how endocrine disrupting chemicals (EDCs), heavy metals, air pollution, and nanoparticles can influence the gut microbiome. The second part of the review will consider how potential environmental chemical-induced gut microbiome changes might subsequently induce pathophysiological responses in the host, although definitive evidence for such effects is still lacking. By understanding how these chemicals result in gut dysbiosis, it may open up new remediation strategies in animals, including humans, exposed to such chemicals.

## Introduction

“*I am large, I contain multitudes*.” When Walt Whitman declared this statement, he meant that each one of us might contradict ourselves by possessing conflicting perspectives and personalities. However, this statement could also aptly describe the fact that almost any large organism, including humans, also involuntarily serves as an incubator for many microorganisms that compete for space and nutrients and can in turn influence host responses, as detailed in Yong's book, “I Contain Multitudes: The Microbes Within Us and a Grander View of Life” (Yong, 2016).

The intestinal flora alone consists of 500 to 1,000 resident species that includes 7,000 to 40,000 bacterial strains representing 1800 genera (Luckey, 1972; Ley et al., 2006a; Frank and Pace, 2008; Qin et al., 2010; Clemente et al., 2012; Douglas-Escobar et al., 2013; Forsythe and Kunze, 2013; Gilbert et al., 2013). While there has been considerable emphasis in characterizing the genome of various animal species, the 10^3^–10^4^ microbiota within the gut collectively have 150 times more genes than the human genome (Gill et al., 2006; Qin et al., 2010). Taken together, the gut microbiome may essentially be considered as a separate “organ” weighing about 1–2 kg that represent 100 trillion individual microrganisms (O'Hara and Shanahan, 2006; Forsythe and Kunze, 2013). With the increasing recognition that microbes may actually serve as the driver for many host responses, the Human Microbiome Project was initiated to understand the complex and bidirectional relationship between host and resident microbiomes (Human Microbiome Project Consortium, 2012a,b).

Past studies have focused on how host health and diet can affect the gut microbiome (Munyaka et al., 2014; Murphy et al., 2015; Xu and Knight, 2015; Zhang and Yang, 2016; Singh et al., 2017). However, other extrinsic factors encountered on a daily basis can exert profound effects on the gut microbiome. Environmental chemicals, including heavy metals, air pollution, nanoparticles, and endocrine disrupting chemicals (EDCs), are increasingly pervasive in terrestrial and aquatic environments, and there is every indication such chemicals will become even more abundant in coming decades (Jurewicz et al., 2013; Yuswir et al., 2013; Caravanos et al., 2014; GrandViewResearch, 2014; Hadrup and Lam, 2014; Theodorou et al., 2014; Chowdhury et al., 2016; Zeng et al., 2016). Such chemicals are also found in everyday items, such as storage containers, plastic water bottles, and antimicrobial materials. Exposure to such chemicals can lead to widespread host effects and also simultaneously target commensal bacteria contained within the gut and possibly other organs. The chemical-induced destruction of the gut flora may open up a Pandora's box leading to disruptions in several host systems, including the central nervous system (CNS) through the gut-microbiome-brain axis (Collins and Bercik, 2009; Rhee et al., 2009; Cryan and Dinan, 2012).

Herein, I will consider the evidence to date that environmental chemicals can lead to gut microbiome disruptions, otherwise termed gut dysbiosis, in various animal species. The gut-microbiome-brain axis will be discussed to illustrate how changes in the gut microbiome may impact health of the host, namely neurobehavioral responses. We will conclude by discussing the unanswered questions and future directions. As we discuss how such chemicals can affect the gut microbiome, it is important to keep a few things in mind. Two types of analyses are commonly described for gut microbiome studies: (1) α-diversity, which refers to the overall diversity within a given sample or group. In contrast, (2) β-diversity compares the bacteria present in individual samples or groups to other samples or groups to determine how much they diverge from each other in the various bacteria or operational taxonomic units (OTUs, microbial organisms, which can be classified at different taxonomic levels). Additionally, most of the recent data discussed below is based on 16s rRNA sequencing approaches. In studies based on on older methods, e.g., targeted PCR or qPCR for specific microorganisms, these are denoted below as a potential limitation in that not all bacterial changes induced by one or more environmental chemicals may have been identified with such approaches.

The studies described below test the effects of environmental chemicals on the gut microbiome in a variety of animal models, such as various strains of mice and rats, zebrafish, and dogs. The underlying assumption is that the gut microbiome in these species resembles that of humans. For rodent models and zebrafish (*Danio rerio*), there are similarities in the overall signature profile of the gut microbiome to humans in health and disease (Ley et al., 2006b; Turnbaugh et al., 2006; Nadal et al., 2009; Santacruz et al., 2009; Borrelli et al., 2016; Liu et al., 2016; Falcinelli et al., 2017; Koo et al., 2017). However, further work and construction of multi-species metagenomic databases, such as MetaPro-IQ, are essential in validating whether various taxa possess analogous resident gut microbes under varying health conditions and environmental fluctuations (Zhang X. et al., 2016).

**Figure 1:**
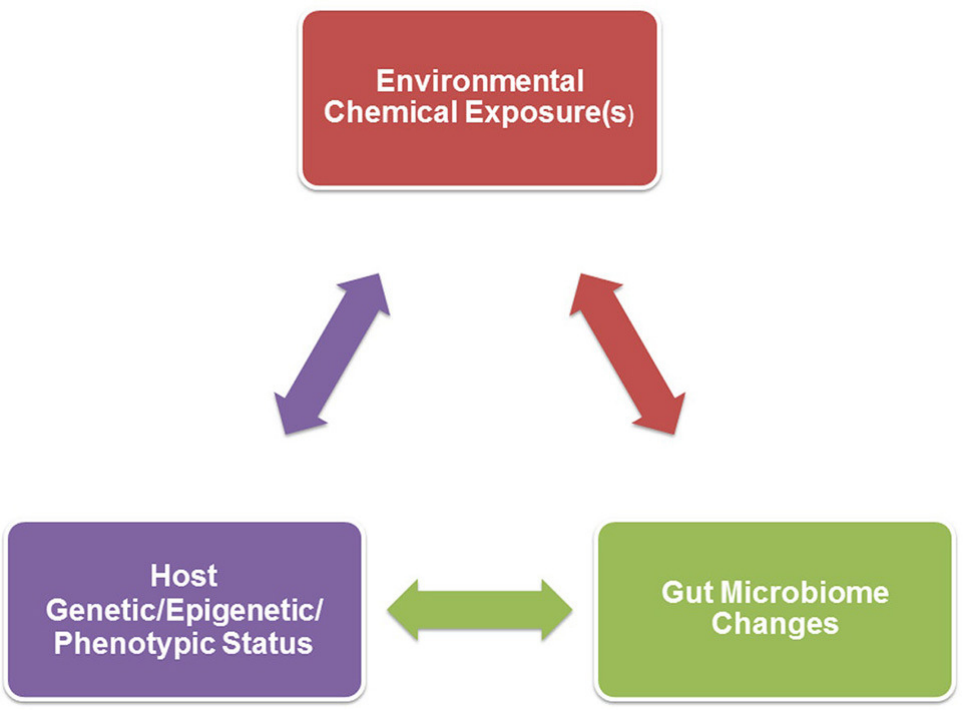
A triad relationship exists between environmental chemical exposure(s), host genetic/epigenetic/phenotypic background, and gut microbiome effects. Environmental chemical-induced host phenotypic changes may result in gut microbiome alterations. Examples of such host changes include hormonal imbalances, obesity, inappetence, gastrointestinal disease, or growth. Environmental toxicants might also directly result in gut dysbiosis that could in turn affect the host, such as neurobehavioral (further detailed in Figure 2), immunological, and metabolic responses. The host genetic/epigenetic/phenotypic status and/or gut microbiome could change the pharmokinetic dynamics of environmental chemicals, including absorption, distribution, metabolism, and/or excretion, which could alter host vulnerability to certain environmental toxicants.

While the studies below have ascribed gut microbiome changes to environmental chemical exposure, many of these chemicals can induce systemic and pathological effects on the host. As illustrated in Figure 1, the gut microbiome changes could thus be secondary to phenotypic changes in the host, including metabolic disorders (obesity/weight loss, inappetence, gastrointestinal disorders, and growth defects to list a few examples). For instance, obesity, starvation, and gastrointestinal disorders could directly affect nutrient substrate availability within the gastrointestinal system and thereby shift proliferation to certain bacterial communities (Sweeney and Morton, 2013; Astbury et al., 2015; Remely et al., 2015; Jonkers, 2016; Nettleton et al., 2016; Hoffman et al., 2017; Seganfredo et al., 2017). Some of the environmental chemicals discussed below, namely bisphenol A (BPA), are associated with inducing metabolic disorders and obesity, and are therefore considered obesogens (Johnson et al., 2015; Janesick and Blumberg, 2016; Heindel et al., 2017). It is not clear though if the metabolic disruptions precede potential gut microbiome changes. As dicussed later, hormones produced by the host can also alter bacterial residents within the gut. Endocrine disrupting chemicals (EDCs) might impact the production of various steroid and peptide hormones. For example, the classic EDC, BPA, can alter production of estrogen, testosterone, glucocorticoids, insulin, and likely other hormones (Akingbemi et al., 2004; Nakamura et al., 2010; Poimenova et al., 2010; Peretz et al., 2011; D'Cruz et al., 2012; Nanjappa et al., 2012; Castro et al., 2013; Garcia-Arevalo et al., 2016; Oliveira et al., 2017; Weldingh et al., 2017). Such EDC-induced endocrinopathies might be another mechanism by which host changes can influence the gut microbiota. To elucidate whether the gut microbiome changes precede host pathophysiological responses or vice versa, repeated assessments of the gut flora and host metabolic state and other responses are essential. However, most of the examples provided below only performed a single assessment of the gut microbiome after direct or developmental exposure to the environmental chemical being considered. Even with these caveats, the studies below provide evidence that exposure to environmental chemicals can alter the compositon of the gut microbiota.

## Effects of heavy metals and particulate matter on the gut microbiome

To date, exposures to heavy metals have been shown to elicit significant effects on the gut microbiome. Six-week-old female C57Bl/6 mice were exposed to 10 ppm arsenic for 4 weeks in the drinking water, whereupon their gut microbiome and metabolome profiles were determined (Lu et al., 2014a). Arsenic exposure resulted in several intestinal flora changes that led to distinct β-diversity clustering of treated vs. control individuals. Specifically, those within the order Streptophyta; family unassigned, order Clostridiales; family unassigned, order Clostridiales; family Catabacteriaceae, order Clostridiales; family Clostridiaceae, and order Erysipelotrichales; family Erysipelotrichaeceae relative abundance was decreased in arsenic-exposed individuals; whereas, order Bacillales; family other and order Clostridiales; family Clostridiales Family XIII Incertae Sedis were increased. Metabolomic analysis also revealed that several metabolites were altered post-arsenic exposure, and these changes were associated with gut microbiome alterations. For instance, indolelactic acid was decreased in this group, which positively correlated with changes in Erhysipelotrichaceae but was negatively associated with Clostridiales Family XIII Incertae Sedis. Arsenic (3 mg/L drinking water), iron (5 mg/L drinking water), and the combination of the two affected the gut microbiome in 5 week old ICR mice exposed to these chemicals for 90 days (Guo et al., 2014). Individuals exposed to one or both metals had an increase in the relative abundance of Firmicutes, Tenericutes, Proteobacteria, but decreases in Bacteroidetes and TM7. Those exposed to arsenic alone showed increases in Acidobacteria and Cyanobacteria/Chloroplast, whereas, Verrucomicrobia was elevated in the iron and iron + arsenic groups. Other genus level changes included an increase in *Lactobacillus* spp. in those exposed to both heavy metals but this bacterium was decreased in individuals exposed to only one of the two metals. Barnesiella and Bacteroides were also reduced in those exposed to only one of the two metals. It is possible that the co-exposure antagonized some of the individual microbial effects of each metal.

In 6 to 8-week-old C57Bl/6 Tac male mice exposed for 2, 5, or 10 weeks to 0, 10, or 250 ppb arsenite (As(III)), time and dose-dependent effects on the gut microbial community were found, especially for Bacteroidetes and Firmicutes (Dheer et al., 2015). Arsenic-treatment removed the bacterial biofilm residing along the mucosal lining and altered the diversity and abundance of microorganisms with bacterial spores increasing and intracellular inclusions reduced with the 250 ppb dose.

Other studies have shown that host genetic status and sex can influence the affects arsenic has on the gut microbiome. Four weeks of arsenic treatment (10 ppm in the drinking water) to wild-type and IL10^−/−^ mice (who can develop colitis depending on the resident gut microbiota) resulted in genetic-dependent gut microbiome changes with an increase in Bacteroidetes but a decrease in Firmicutes in arsenic-treated IL10^−/−^ mice (Lu et al., 2014b). The gut microbiome alterations in these transgenic mice were linked with reduced ability to detoxify inorganic arsenic. However, no histological differences in the intestines were observed in relation to genetic status or arsenic-treatment. This same arsenic dose and treatment duration underpinned sex-dependent changes in the gut microbiome of C57/BL6 mice (Chi et al., 2016). This treatment induced greater number of bacterial alterations in females with *Dorea* spp. decreasing but *Akkermansia* spp. significantly increasing. Males had an increase in *Dorea* spp. but no changes in relative abundance of *Akkermansia* spp. Correlations between arsenic-induced gut microbiome changes and metabolic pathways involved in metal resistance (including mercury resistance operon, zinc resistance, and the mdtABCD multidrug resistance cluster) and cell transport system (such as glutathione-regulated potassium-efflux system, ATP-dependent efflux pump transporter Ybh, general secretion pathway, and iron acquisition in *Streptococcus*) were increased in females. In treated males, the gut microbiome changes were associated with nitrogen, carbon, and sulfate metabolism.

Lead (Pb) is another heavy metal that has re-emerged as a growing concern with the Flint Drinking Water Crisis (Hanna-Attisha et al., 2016; Nelson, 2016; Heard-Garris et al., 2017; Rosen et al., 2017). However, Pb can be present in a wide range of items and has been previously used in gasoline as tetraethyl lead. Potential sources include water, air, diet, and old paint cans resulting in various routes of exposure, such as ingestion, inhalation, and transdermal. The Center for Disease Control and Prevention (CDC) has determined that there is no safe blood level for Pb (Betts, 2012). This agency has lowered the blood lead level (BLL) of concern from 10 to 5 μg/dl (Betts, 2012), but even lower levels may elicit disease (Gilbert and Weiss, 2006). The studies below testing the effects of Pb on the gut microbiome in rodent models may thus all be considered physiologically and environmentally relevant.

Non-agouti (*a/a*) offspring derived from *A*^*vy*^*/a* male mice bred to *a/a* female mice exposed from gestation through lactation to Pb (32 ppm in the drinking water) undergo shifts in gut microbiota populations with Bacteroidetes and Firmicutes inversely associated with maternal Pb exposure. Cultivable aerobes decreased but anaerobes increased in the Pb-exposed offspring. Intestinal flora changes were associated with an increase in adult body weight in males but not females (Wu et al., 2016).

Exposure of 6-week-old Balb/C female mice for 8 weeks to lead (PbCl_2_, 100 or 500 ppm- mg/L) or cadmium (CdCl_2_, 20 or 100 ppm- mg/L) in the drinking water reduced the numbers of Lachnospiraceae but elevated the relative amounts of Lactobacillaeceae and Erysipelotrichaeceacae with the latter primarily being due to changes in *Turicibacter* spp. (Breton et al., 2013). Another study that exposed adult C57Bl/6 female mice to 10 ppm PbCl_2_ in the drinking water for 13 weeks for a concentration of ~2 mg/kg body weight/day revealed that this chemical altered the gut microbiome trajectory and phylogenetic diversity; gut metabolic disruptions were also determined when fecal samples were assessed at 4 and 13 weeks post-exposure (Gao et al., 2017). With age, the phylogenetic diversity of the gut microbiome increased in controls, but this response was blunted in PbCl_2_-treated animals with Clostridiales, *Ruminococcus* spp., Ruminococcaceae, *Oscillospira* spp. relative amounts decreased in this group. Metabolic pathways involving vitamin E, bile acids, nitrogen metabolism, energy metabolism, oxidative stress, and defense/detoxification were potentially altered in treated individuals.

Heavy metal pollution might influence the gut microbiome in wild populations that are chronically being exposed to such chemicals in aquatic and terrestrial environments. To test this notion, the profiles of two populations of Mongolian toads (*Bufo raddei*) were compared with one living in a heavy-metal-polluted area (Baiyin-BY) and the other residing in a relatively unpolluted area (Liujiaxia-LJX) (Zhang W. et al., 2016). Those inhabiting the BY area had an overabundance of Bacteroidetes; whereas, Tenericutes were over-represented in those from LJX. In the BY toads, the ratio of Firmicutes/Bacteroidetes and the proportion of beneficial bacteria in the gut microbiome were decreased relative to LJX toads. Species diversity and proportion of OTUs) were also reduced in the toads subjected to heavy metal pollution.

To determine how particulate matter (PM), a key pollutant in ambient air, affect the intestines and gut microbiome, wild-type (WT) 129/SvEv mice were orally gavaged with Ottawa urban PM10 (EHC-93: 18 μg/g/day) for 7 or 14 days, and to assess longer term effects of exposure, IL10 deficient (−/−) mice were subjected to the same treatment for 35 days (Kish et al., 2013). WT mice exposed to PM10 for a short duration had alteration in immune gene expression, enhanced pro-inflammatory cytokine secretion into the small intestine, increased gut leakiness (permeability), and hyporesponsiveness in splenocytes to the PM. In IL10^−/−^ mice exposed for a longer duration, increased pro-inflammatory cytokine expression occurred in the colon, and these animals demonstrated significant changes in the relative amounts of *Bacteroidetes* spp., *Firmicutes* spp., and *Verrucomicrobia* spp. Treated mice also showed bacterial-associated changes in short chain fatty acid (SCFA) production with increased abundance of branched chain fatty acids- isobutyrate and isovalerate in the cecum.

The above articles provide evidence that exposure to heavy metals and particulate matter can alter the gut microbiome in a various species, especially mice and rats. However, for the most part, the bacterial changes vary across studies. The few exceptions are heavy metals and heavy metal pollution in rodents and toads appears to alter Bacteroidetes and Firmicutes, but the directionality for both differs across studies (Guo et al., 2014; Lu et al., 2014b; Wu et al., 2016; Zhang W. et al., 2016). The relative abundance of Tenericutes was increased in ICR mice exposed to arsenic and Mongolian toads residing in a heavy-metal polluted area (Guo et al., 2014; Zhang W. et al., 2016).

The conflicting data on which bacteria are altered after heavy metal exposure might be explained by several factors. As revealed in Table 1, many of the current studies have employed mouse models that range in strain, genotype, and even epigenetic status. The diverging studies have tested differing chemicals and doses. Genetic background of the animal might interact with environmental chemical(s) (G × E interaction) to influence the net gut microbiome changes. There could also be three-way interactions between genetics of the host, environmental chemical exposure, and resident gut microbiome (G × E × GM), as illustrated in Figure 1.

**Table 1:** Studies linking environmental chemical exposure and gut microbiome changes.

**Publication**	**Animal model**	**Dosing regimen**	**Major gut microbiome and host findings**
**HEAVY METALS AND PARTICULATE MATTER**			
Lu et al., 2014a	6-week-old C57Bl6/6 female mice	Mic were treated with 10 ppm arsenic in the drinking water for 4 weeks.	•Arsenic exposure led to distinct β-diversity clustering in the intestinal flora. •Relative abundance of bacteria within the order Streptophyta; family unassigned, order Clostridiales; family unassigned, order Clostridiales; family Catabacteriaceae, order Clostridiales; family Clostridiaceae, and order Erysipelotrichales; family Erysipelotrichaeceae were decreased in arsenic-exposed individuals. •Relative abundance of bacteria within the order Bacillales; family other and order Clostridiales; family Clostridiales Family XIII Incertae Sedis were increased. •Indolelactic acid was decreased in arsenic-treated group, which positively correlated with changes in Erhysipelotrichaceae but negatively associated with Clostridiales Family XIII Incertae Sedis.
Guo et al., 2014	5-week-old ICR mice	Mice were treated with arsenic (3mg/L in drinking water), iron (5mg/L in drinking water) or both treatments at the same dosages for 90 days.	•All individuals exposed to one or both metals had an increase in the relative abundance of Firmicutes, Tenericutes, Proteobacteria, but decreases in Bacteroidetes and TM7. •Arsenic-treated mice had increases in Acidobacteria and Cyanobacteria/Chloroplast; •Verrucomicrobia was elevated in the iron and iron + arsenic groups. •*Lactobacillus* spp. was increased in those exposed to both heavy metals but decreased in individuals exposed to only one of the two metals. Barnesiella and Bacteroides were also reduced in those exposed to only one of the two metals.
Dheer et al., 2015	6–8 week-old C57Bl/6 Tac male mice	Mice were exposed for 2, 5, 19 weeks to 0, 10, or 250 ppb arsenite As(III).	•As(III) altered the gut microbial community, in particular for Bacteroidetes and Firmicutes. •Arsenic-treatment removed the bacterial biofilm residing along the mucosal lining •This treatment disrupted the diversity and abundance of microorganisms with bacterial spores increasing and intracellular inclusions reduced with the 250 ppb dose.
Lu et al., 2014b	Wild-type (WT) and IL10^−/−^ mice	Mice were exposed to 10 ppm arsenic in the drinking water for 4 weeks.	•An increase in Bacteroidetes but a decrease in Firmicutes occurred in arsenic-treated IL10^−/−^ mice. •These mice also had reduced ability to detoxify inorganic arsenic. •No histopathological changes were evident in the intestines due to genetic status or arsenic-treatment.
Chi et al., 2016	C57/BL6 male and female mice	Mice were exposed to 10 ppm arsenic in the drinking water for 4 weeks.	•Arsenic exposure induced more gut microbiome alterations in females. •In arsenic treated females, *Dorea* spp. decreased but *Akkermansia* spp. significantly increased. •Arsenic-treated males had an increase in *Dorea* spp. •In females, arsenic-induced gut microbiome changes correlated with metabolic pathways involved in metal resistance (including mercury resistance operon, zinc resistance, and the mdtABCD multidrug resistance cluster) and cell transport system (such as glutathione-regulated potassium-efflux system, ATP-dependent efflux pump transporter Ybh, general secretion pathway, and iron acquisition in *Streptococcus*). •In treated males, the gut microbiome changes were associated with nitrogen, carbon, and sulfate metabolism.
Wu et al., 2016	Non-agouti (*a/a*) mouse offspring	Female (*a/a*) mice were exposed from gestation through lactation to Pb (32 ppm in the drinking water), which resulted in a maternal blood lead level (BLL) of 32 μg/dL.	•Bacteroidetes and Firmicutes in the offspring gut microbiome were inversely associated with maternal Pb exposure.
			•Cultivable aerobes decreased but anaerobes increased in the Pb-exposed offspring. •Intestinal flora changes were associated with an increase in adult body weight in male but not female offspring.
Breton et al., 2013	6-week-old Balb/C female mice	Mice were exposed for 8 weeks to lead (PbCl_2_, 100 or 500 ppm- mg/L) or cadmium (CdCl_2_, 20 or 100 ppm- mg/L) in the drinking water.	•Exposure to either heavy metal reduced the numbers of Lachnospiraceae but elevated the relative amounts of Lactobacillaeceae and Erysipelotrichaeceacae with the latter primarily being due to changes in *Turicibacter* spp.
Gao et al., 2017	Adult C57Bl/6 female mice	Mice were exposed to 10 ppm PbCl_2_ in the drinking water for 13 weeks to a concentration of ~2 mg/kg body weight/day.	•PbCl_2_-treated animals did not show typical age-dependent increase in phylogenetic diversity. •Clostridiales, *Ruminococcus* spp., Ruminococcaceae, *Oscillospira* spp. decreased in treated mice. •Metabolic pathways involving vitamin E, bile acids, nitrogen metabolism, energy metabolism, oxidative stress, and defense/detoxification were disrupted in PbCl_2_-exposed mice.
Zhang W. et al., 2016	Mongolian toads (*Bufo raddei*)	The gut microbiome was assayed in toads living in a heavy-metal-polluted area (Baiyin- BY) and compared to those living in a relatively unpolluted area (Liujiaxia- LJX).	•Toads in the BY area had an overabundance of Bacteroidetes. •Tenericutes were over-represented in those from LJX. •In the BY toads, the ratio of Firmicutes/Bacteroidetes and the proportion of beneficial bacteria in the gut microbiome were decreased relative to LJX toads. •Species diversity and proportion of OTUs were also reduced in the toads subjected to heavy metal pollution.
Kish et al., 2013	WT 129/SvEv &IL10^−/−^ mice	WT 129/SvEv mice were orally gavaged with Ottawa urban PM10 (EHC-93: 18 μg/g/day) for 7 or 14 days. Longer-term effects of PM10 exposure were assessed in IL10^−/−^ mice who were subjected to the same treatment for 35 days.	•WT mice exposed to PM10 had alterations in immune gene expression, enhanced pro-inflammatory cytokine secretion into the small intestine, increased gut leakiness (permeability), and hyporesponsiveness in splenocytes to the PM. •IL10^−/−^ mice exposed to PM10 had increased pro-inflammatory cytokine expression in the colon and significant changes in the relative amounts of *Bacteroidetes* spp., *Firmicutes* spp., and *Verrucomicrobia* spp. •The treated IL10^−/−^ mice had bacterial-associated changes in SCFA production with increased abundance of branched chain fatty acids- isobutyrate and isovalerate in the cecum.
**NANOPARTICLES**			
Williams et al., 2015	Male and female Sprague-Dawley rats	Rats were exposed orally for 13 weeks to various sizes (10, 75, and 110 nm) and doses (9, 18, and 36 mg/kg body weight/day) of AgNPs.	•AgNP-exposed rats had decreased populations of Firmicutes and *Lactobacillus* but greater proportion of potentially-pathogenic gram negative bacteria. •Rats treated with the lower doses and sizes of AgNPs showed increased intestinal-mucosal gene expression of immunomodulatory genes, *Muc3, Tlr2, Tlr4, Gpr43*, and *Foxp3*.
van den Brule et al., 2016	C57Bl/6 female mice	Mice were dosed orally for 28 days to AgNPs (0, 46, 460, 4,600 ppb).	•Bacterial sequences and populations in the gut microbiome changed in a dose-dependent manner with AgNP exposure.•AgNPs increased the ratio between Firmicutes (F) and Bacteroidetes (B) phyla because of changes in the distribution of Lachnospiraceae and the S24-7 family, respectively.
Javurek et al., 2017	Male Sprague-Dawley rats	Rats were exposed for 2 weeks to AgNPs (3.6 mg/kg body weight) in two forms: cubes and spheres.	•*Clostridium* spp., *Bacteroides uniformis*, Christensenellaceae, and *Coprococcus eutactus* were reduced in AgNC exposed rats. •*Oscillospira* spp., *Dehalobacterium* spp., Peptococcaeceae, *Corynebacterium* spp., *Aggregatibacter pneumotropica* were suppressed in the AgNS exposed individuals. •The gut microbiome changes correlated with behavioral responses observed when the same rats were tested in EPM.
Han et al., 2014	Fruit fly (*Drosophila melanogaster*) larvae	Larvae were exposed to AgNPs (50 μg/ml) or CuNPs (50 μg/ml).	•Larvae exposed to AgNPs show a less diverse gut microbiota, overgrowth of *Lactobacillus brevis* but a decrease in Acetobacter relative to controls or those exposed to CuNPs.
Merrifield et al., 2013	Zebrafish (*Danio rerio*)	Zebrafish were fed diets laced with AgNPs or CuNPs (500 mg/kg food for 14 days).	•Select beneficial microbes, e.g. *Cetobacterium somerae*, were reduced to undetectable levels in those exposed to CuNPs. •Two uncharacterized bacteria within the Firmicutes phylum were suppressed by CuNPs but not AgNPs. •CuNPs led to greater gut microbiome changes than AgNPs, although some OTUs were sensitive to AgNPs. •Neither type of NPs affected the intestinal epithelial lining of exposed zebrafish.
Sarkar et al., 2015	Early fingerlings of tilapia (*Oreochromis nilticus L*.)	Early fingerlings of tilapia were exposed to two sublethal concentrations of AgNPs (0.4 and 0.9 mg/L for 21 days).	•Histopathological analysis of the intestines showed reduced thickness of the intestinal wall, mucosal swelling, and increased catalase expression in AgNP-treated fish. •The overall amount of gut microflora (α-diversity) was reduced in a dose-dependent manner with AgNP exposure, which was accompanied by increase in glutamate dehydrogenase activity.
Das et al., 2014	Gut bacteria from a human donor	A defined bacterial community from a healthy human donor was subjected to 48 h of exposure to AgNPs (25, 100, and 200 mg/L).	•AgNP resulted in a negative influence on bacterial communities, as measured by gas production and changes in fatty acid methyl ester profiles. •AgNPs also induced bacterial community changes with *Bacteroides ovatus, Roseburia faecalis, Eubacterium rectale, Roseburia intestinalis*, and *Ruminococcus torques* significantly reduced. •*Raoultella* spp., *Escherichia coli* were increased after exposure to the various concentrations of AgNPs.
Yausheva et al., 2016	Redworms (*Eisenia fetida*)	ZnNPs were added at 1,000 mg/kg to the substrate soil provided to redworms.	•ZnNPs in the soil led to an increase in worm mortality rate (35%). •Exposure to ZnNPs also affected the gut microbiome within the worms with a reduction in β-diversity (303 OTUs in controls vs. 78 OTUs in treated individuals). •While Firmicutes was decreased in ZnNP-treated worms, there were overgrowths of Proteobacteria (primarily due to increases in *Verminephrobacter* spp. and *Ochrobactrum* spp.) in exposed individuals.
**ENDOCRINE DISRUPTING CHEMICALS (EDCs)**			
Liu et al., 2016	Adult male zebrafish	Zebrafish were exposed for 5 weeks to BPA (200 μg/L or 2,000 μg/L) or E2 (500 ng/L or 2,000 ng/L).	•BPA or E2 exposure resulted in increased hepatic expression of vitellogenin expression. •BPA or E2 exposed zebrafish also had changes in composition of the intestinal flora with CKC4 increasing significantly.
Javurek et al., 2016	Adult male and female California mice (*Peromyscus californicus*) Juvenile (PND 30) male and female California mice offspring	Female California mice were exposed 2 weeks prior to mating to BPA (50 mg/kg feed weight), EE (0.1 ppb), or a control diet, and they were continued on the diets throughout gestation and lactation (PND 30). After pairing, reproductive male partners were exposed to these diets until offspring were weaned at PND 30. Male and female offspring were exposed to these above chemicals through the maternal diet and milk from periconception through lactation, respectively.	•Exposure to BPA and EE resulted in generational and sex-dependent gut microbiome changes. •Several of the bacteria whose relative abundance increased with BPA or EE exposure in the P_0_ or F_1_ generation, namely *Bacteroides* spp., Mollicutes, Prevotellaceae, Erysipelotrichaceae, Akkermansia, Methanobrevibacter, *Sutterella* spp., are associated with various diseases, such as inflammatory bowel disease (IBD), metabolic disorders, and colorectal cancer. •The relative abundance of the beneficial bacterium, *Bifidobacterium* spp., was elevated in fecal samples of BPA- and EE-exposed F_1_ females. •Gut microbiota alterations were also associated with alterations in various metabolic and other pathways.
Lai et al., 2016	3-week-old CD1 male mice	Mice were subjected for 10 weeks to one of these three treatments: BPA (120 μg/ml in the water), sucrose water solution (high sucrose diet- HSD, 200 mg/ml), or consumption of a high fat diet (HFD).	•Cecal contents from these three groups were similar in α- and β-diversity in terms of gut microbial community structure. •Both the BPA and HFD groups had relative overabundance of Proteobacteria •The BPA and HFD groups also had relative increases in Helicobacteraceae but reductions in relative abundance of Firmicutes and *Clostridal* spp.
Koestel et al., 2017	Adult dogs (*Canis familiaris)*	Adult gonadectomized male and female dogs were switched from being fed dry dog food and placed for 2 weeks on one of two brands of commercial canned dog food.	•After 2 weeks of being on either commercial brand canned dog food, dogs in this study had an increase of BPA concentrations by almost three-fold. •Relative abundance of *Bacteroides* spp., Streptophyta, Erysipelotrichaceae, and *Flexispira* spp. negatively correlated with greater circulating levels of BPA. •*Bacteroides ovatus, Prevotella* spp., *Ruminococcus* spp., and *Cetobacterium somerae* positively correlated with elevated concentrations of BPA.
Hu et al., 2016	Sprague-Dawley female rats	Sprague-Dawley female rats were chronically exposed from birth to adulthood to diethyl phthalate (DEP- 0.1735 mg/kg body weight), methylparaben (MPB- 0.1050 mg/kg body weight), triclosan (TCS- 0.05 mg/kg body weight), or the mixture of these three chemicals.	•Chronic exposure to these individual or combined chemicals led to microbiome changes by adolescence but many of these changes were abolished by adulthood •The changes observed in adolescence included the relative abundance of Bacteroidetes (*Prevotella* spp.) was increased but Firmicutes (*Bacilli* spp.) was reduced in all treated groups. •Rats treated with DEP or MPB showed reduced body weight at adolescence.

Several of the above studies also exposed individuals to the heavy metal via the drinking water, which is generally considered a reasonable method that replicates dietary exposure to such chemicals in humans. However, we may also be exposed to such chemicals through other avenues, including inhalation or possibly transdermal. It is not clear though whether such other routes of exposure result in gut microbiome changes. Most of the current studies only tested a single dose for varying lengths of time. To better understand how heavy metals effect the gut microbiome, varying doses that recapitulate human exposure under varying conditions (such as those individuals living in heavily polluted areas to those with relatively minimal exposure) should be tested, along with differing intervals of time to span acute to chronic exposure effects. Lastly, the above studies generally used 16s rRNA sequencing, which generally provides sufficient coverage and sensitivity to detect differences between treatments. However, the bioinformatic analyses, namely quantitative assessments, are important when attempting to compare across studies. In moving forward, it would be helpful if agencies funding environmental chemical studies, such as the US National Institute of Environmental Health Sciences (NIEHS) provide funding for creation of a bioinformatics workflow management database where investigators can employ uniform methods to analyze and eventually deposit metagenomics data derived from environmental chemical exposures. The resulting database could then be searchable by other users in the field.

## Effects of nanoparticles on the gut microbiome

Current rodent studies have yielded conflicting results whether silver nanoparticles (AgNPs) and other nanoparticles affect the gut microbiome (Hadrup et al., 2012; Merrifield et al., 2013; Song et al., 2015; Wilding et al., 2015; Williams et al., 2015; Frohlich and Frohlich, 2016; van den Brule et al., 2016).

Male and female Sprague-Dawley rats exposed orally for 13 weeks to various sizes (10, 75, and 110 nm) and doses (9, 18, and 36 mg/kg body weight/day) of AgNPs show decreased populations of Firmicutes and *Lactobacillus* but greater proportion of potentially-pathogenic gram negative bacteria (Williams et al., 2015). The lower doses and sizes of AgNPs also suppressed intestinal-mucosal gene expression of immunomodulatory genes, *Muc3, Tlr2, Tlr4, Gpr43*, and *Foxp3*. One limitation of this study was that it used real-time PCR analysis to screen select bacterial groups. By using a global approach (16S rRNA sequencing) of gut bacteria from mice exposed to similar sizes, doses, and duration of AgNPs, another study reported that these treatments did not alter the gut microbiome (Wilding et al., 2015). Measurement of cecal bacterial phyla from 4-week-old rats treated with varying doses of AgNPs for 28 days also did not detect any bacterial differences post-exposure (Hadrup et al., 2012).

However, experiments with next generation sequencing (NGS) found that C57Bl/6 female mice dosed orally for 28 days to AgNPs (0, 46, 460, 4,600 ppb) exhibited changes in the relative abundance of bacteria that depended on the exposure dose (van den Brule et al., 2016). Further, AgNPs increased the ratio between Firmicutes (F) and Bacteroidetes (B) phyla likely due to changes in the distribution of Lachnospiraceae and the S24-7 family, respectively.

In our recent study with male Sprague-Dawley rats exposed for 2 weeks to AgNPs (3.6 mg/kg body weight) in two forms: cubes and spheres, gut microbiota changes varied according to shape of the AgNPs (Javurek et al., 2017). *Clostridium* spp., *Bacteroides uniformis*, Christensenellaceae, and *Coprococcus eutactus* were reduced in the AgNC exposed rats. In contrast, *Oscillospira* spp., *Dehalobacterium* spp., Peptococcaeceae, *Corynebacterium* spp., *Aggregatibacter pneumotropica* were suppressed in AgNS exposed individuals. The gut microbiome changes also correlated with behavioral responses observed when the same animals were examined in the elevated plus maze (EPM), a test designed to measure anxiety-like and exploratory behaviors in rodents.

The effects of AgNPs and other NPs have been assessed in other species. AgNPs may be a potential antimicrobial additive in pigs (Fondevila et al., 2009). Fruit fly (*Drosophila melanogaster*) larvae exposed to AgNPs (50 μg/ml) show a less diverse gut microbiota, overgrowth of *Lactobacillus brevis* but a decrease in Acetobacter relative to controls or those exposed to copper (Cu) NPs (50 μg/ml) (Han et al., 2014).

In zebrafish (*D. rerio*), feeding of diets laced with AgNPs or CuNPs (500 mg/kg food for 14 days) changed the composition of the intestinal microbiome (Merrifield et al., 2013). Select microbes, e.g., *Cetobacterium somerae* were reduced to unmeasurable levels in those exposed to CuNPs, and two uncharacterized bacteria within the Firmicutes phylum were inhibited by CuNPs but not AgNPs. Overall, CuNPs stimulated greater gut microbiome changes than AgNPs in this study, although some OTUs were sensitive to AgNPs. Neither type of NPs affected the intestinal epithelial lining of exposed zebrafish. Early fingerlings of tilapia (*Oreochromis nilticus L*.) exposed to one of two sub-lethal concentrations of AgNPs (0.4 and 0.9 mg/L for 21 days) demonstrated pathological changes in the intestines and gut microbiome disruptions (Sarkar et al., 2015). Histopathological analysis of the intestines showed reduced thickness of the intestinal wall, mucosal swelling, and increased catalase expression in AgNP-treated fish. The overall amount of gut microflora was reduced by AgNPs in a dose-dependent manner, which was accompanied by an increase in glutamate dehydrogenase activity.

To determine whether AgNPs could affect the gut microbiome composition in humans, one study examined the effects of 48 h of exposure to AgNPs (25, 100, and 200 mg/L) on a defined bacterial community established from a healthy human donor (Das et al., 2014). Their findings reveal that these particles resulted in a shift to more pathogenic bacterial species, as measured by gas production and changes in fatty acid methyl ester profiles. AgNPs also induced bacterial community changes with *Bacteroides ovatus, Roseburia faecalis, Eubacterium rectale, Roseburia intestinalis*, and *Ruminococcus torques* significantly decreased but *Raoultella* spp., *Escherichia coli* increased after exposure to the various concentrations of AgNPs.

Addition of zinc nanoparticles (ZnNPs) at 1,000 mg/kg to the substrate soil provided to redworms (*Eisenia fetida*) led to an increase in mortality rate (35%) in this group (Yausheva et al., 2016). Exposure to ZnNPs also affected the worm's gut microbiome with a reduction in α-diversity (303 OTUs in controls vs. 78 OTUs in treated individuals). While Firmicutes was decreased in ZnNP-treated worms, there were overgrowths of Proteobacteria (primarily due to increases in *Verminephrobacter* spp. and *Ochrobactrum* spp.) in exposed individuals.

Similar to results obtained with heavy metals, the collective studies to date for nanoparticle exposure in various animal models and systems that include mice, rats, fruit flies, zebrafish, tilapia, redworms, and gut bacteria from a human donor, there is discordance across studies as to which gut microbiota are altered by these environmental chemicals. Firmicutes and *Lactobacillus* spp. are the only ones affected in two or more of the studies listed above that represent various taxa (rats, mice, fruit flies, zebrafish, and redworms) (Merrifield et al., 2013; Williams et al., 2015; van den Brule et al., 2016; Yausheva et al., 2016). All of the above studies used a global approach to screen for gut bacterial changes, except for Williams et al. (2015) that used a real-time PCR method to identify select bacteria. The same explanations and potential solutions exists to account for differences in gut microbiome changes identified after exposure to varying nanoparticles. Additionally, the collective findings with nanoparticle exposure represent extremely diverse species and model systems, which may complicate cross-study comparisons. Even so, two of the same bacterial changes were identified across taxa exposed to nanoparticles (as detailed above). By testing effects in multiple taxa, it could also reveal those gut microbes that are the most sensitive to certain environmental chemicals regardless of their host species.

## Effects of endocrine disrupting chemicals on the gut microbiome

Adult and developmental exposure to bisphenol A (BPA), estradiol (E2) or ethinyl estradiol (EE, estrogen in birth control pills) can affect the gut microbiome in rodent models, dogs, and zebrafish (Javurek et al., 2016; Lai et al., 2016; Liu et al., 2016; Koestel et al., 2017). Exposure of adult male zebrafish for 5 weeks to BPA (200 or 2,000 μg/L) or E2 (500 ng/L or 2,000 ng/L) increased hepatic expression of vitellogenin expression (a biomarker of estrogen exposure in male fish), and restructured the intestinal flora with those within the CKC4 phylum increasing significantly (Liu et al., 2016).

Adult female P_0_ California mice (*Peromyscus californicus*) were exposed to two weeks prior to mating to BPA (50 mg/kg feed weight), EE (0.1 ppb), or a control diet, and then continued on the diets throughout gestation and lactation (post-natal day- PND 30) (Javurek et al., 2016). This species is monogamous and biparental, and thus, their P_0_ male breeding partner was also consuming these diets from the time of mating through PND 30. At PND 30 (weaning), the gut microbiome of the P_0_ parents and F_1_ male and female offspring was analyzed. Exposure to BPA and EE resulted in generational and sex-dependent gut microbiome changes. Several of the bacteria whose relative abundance increased with BPA or EE exposure in the P_0_ or F_1_ generation, namely *Bacteroides* spp., Mollicutes, Prevotellaceae, Erysipelotrichaceae, Akkermansia, Methanobrevibacter, *Sutterella* spp., are associated with various diseases, such as inflammatory bowel disease (IBD), metabolic disorders, and colorectal cancer. The relative abundance of the bacterium, *Bifidobacterium* spp., was elevated in fecal samples of BPA- and EE-exposed F_1_ females. Gut microbiota alterations were also associated with alterations in various metabolic and other pathways.

Three-week-old male CD1 mice were subjected for 10 weeks to one of these three treatments: BPA (120 μg/ml in the water), sucrose water solution (high sucrose diet- HSD, 200 mg/ml), or consumption of a high fat diet (HFD) (Lai et al., 2016). Analysis of the cecal contents from the three groups relative to controls indicated that all three treatments mediated similar α- and β-diversity in changes of gut microbial community structure. Both the BPA and HFD groups had relative overabundance of Proteobacteria, which may be considered a microbial marker for dysbiosis. These two groups also had relative increases in Helicobacteraceae but reductions in relative abundance of Firmicutes and *Clostridal* spp.

Adult gonadectomized male and female dogs (*Canis familiaris)* switched from being fed dry dog food to being placed for two weeks on one of two brands of commercial canned dog food had an increase in circulating BPA concentrations by almost three-fold (Koestel et al., 2017). Relative abundance of eight bacteria were associated with serum BPA concentrations in dogs feed either diet. *Bacteroides* spp., Streptophyta, Erysipelotrichaceae, and *Flexispira* spp. negatively correlated with greater circulating levels of BPA. However, *B. ovatus, Prevotella* spp., *Ruminococcus* spp., and *Cetobacterium somerae* were positively associated with elevated concentrations of BPA.

Chronic exposure of Sprague-Dawley female rats from birth through adulthood to diethyl phthalate (DEP- 0.1735 mg/kg body weight), methylparaben (MPB- 0.1050 mg/kg body weight), triclosan (TCS- 0.05 mg/kg body weight), or the mixture of these three chemicals led to microbiome changes by adolescence but many of these changes were diminished by adulthood (Hu et al., 2016). The changes observed in adolescence included the relative abundance of Bacteroidetes (*Prevotella* spp.) was increased but Firmicutes (*Bacilli* spp.) was reduced in all treated groups. Those treated with DEP or MPB showed reduced body weight at adolescence.

The effects of the EDCs, BPA and phthalates, on the gut microbiome have been examined to date in zebrafish, California mice, CD1 mice, dogs, and Sprague-Dawley rats. While there are differences across studies in which bacteria are affected, the combined studies reveal select gut microbes that are affected across species. Firmicutes was increased in BPA-exposed CD1 mice and rats exposed to DEP, MPB, TCS, or the mixture of these chemicals (Hu et al., 2016; Lai et al., 2016). Relative abundance of *Bacteroides* spp. was elevated in BPA-exposed California mice and dogs with greater circulating levels of BPA (Javurek et al., 2016; Koestel et al., 2017). Prevotellaceae/*Prevotella* spp. was increased in BPA-exposed California mice, dogs with increased levels of BPA, and rats exposed to DEP, MPB, TCS, or the mixture of these chemicals (Hu et al., 2016; Javurek et al., 2016; Koestel et al., 2017). All of the above studies used a global approach to identify bacterial differences. Possible differences across studies and method that can be used to reconcile conflicting analytical data are the same as detailed for heavy metals and nanoparticles.

## Gut microbiome changes and secondary host effects

It is increasingly becoming apparent that gut microbiome disruptions can contribute to many host diseases, as depicted in Figure 1. However, it is beyond the scope of the current review to consider all of the mechanisms by which gut dysbiosis impacts host health. Thus, we will consider one well-recognized axis: the gut-microbiome-brain axis to illustrate some examples of how even small changes in microbial communities can be associated with severe host disease in the form of neurobehavioral disorders, such as autism spectrum disorders (ASD).

### Gut-microbiome-brain axis

**Figure 2:**
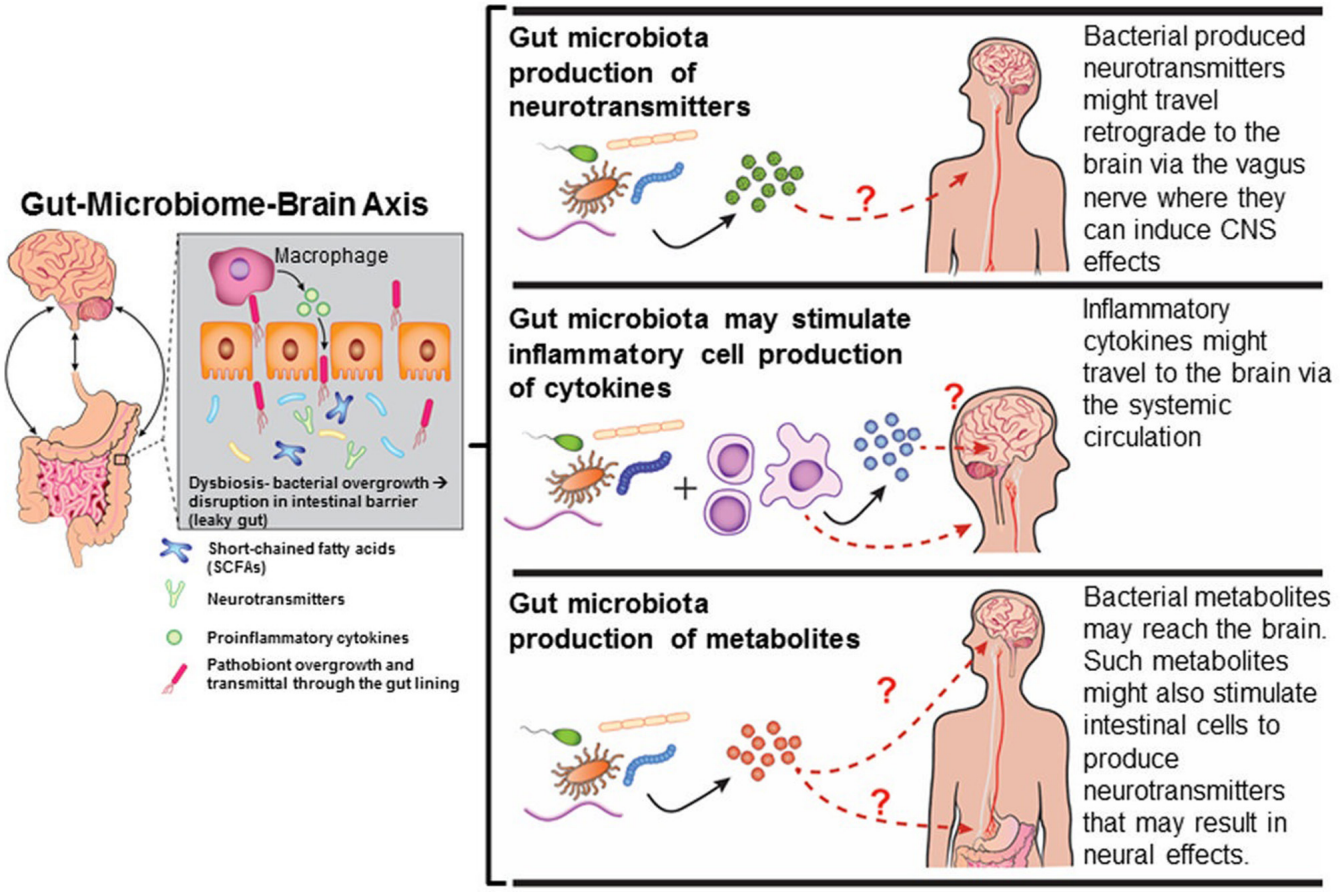
Mechanisms by which gut dysbiosis may result in neurobehavioral disorders. Diagram has been drawn based on Figures in http://sitn.hms.harvard.edu/flash/2016/second-brain-microbes-gut-may-affect-body-mind/; Borre et al., 2014.

Several mechanisms exist by which gut dysbiosis may influence neurobehavioral responses (Figure 2) and as reviewed in Rosenfeld (2015). Tight junctions between the enterocytes typically prevent bacteria from accessing the underlying mucosal blood vessels with an estimate of greater than 100 tons of food-borne factors, including microorganisms, processed in a single lifetime (Alonso et al., 2014). With each turning over of epithelial cells, the barrier has to be continually re-established and overgrowth of bacterial pathogens or indigenous pathobionts harbored within the gut can use various strategies to disrupt this barrier, and thereby, facilitate an increase in “gut leakiness” (Ashida et al., 2011). Under these conditions, bacteria, antigens, virulence factors, and bacterial metabolites can penetrate through the intestinal lining and invade into the underlying blood vessels. Bacterial metabolites and virulence factors might pass through the blood-brain-barrier. This transference due to a “leaky gut” may play a crucial role in many gut-microbiome-brain comorbidity disorders (Ait-Belgnaoui et al., 2012; Alonso et al., 2014).

It has long been recognized that the brain regulates gastrointestinal function via the enteric nervous system (ENS) and vagal nerve (Reviewed in Mayer, 2011). Discovery that absence of a gut microbiome, as occurs in gnobiotic or germ-free (GF) mice, results in various behavioral disruptions (Sudo et al., 2004; Diaz Heijtz et al., 2011; Gareau et al., 2011; Neufeld et al., 2011; Clarke et al., 2013; Desbonnet et al., 2014), shifted the focus of attention as to how gut microbiota might affect neural responses (Reviewed in Douglas-Escobar et al., 2013; Stilling et al., 2014; Rosenfeld, 2015). Synaptogenetic proteins (synaptophysin and PSD-95) are suppressed in GF mice (Diaz Heijtz et al., 2011). Vagotomy or chemical sypathectomy alleviates the behavioral changes observed in GF mice, suggesting that the vagal nerve serves as an important conduit between the gut and brain responses (Bercik et al., 2011).

Bacterial metabolites might also trigger neurobehavioral responses or induce encephalopathic effects. Some metabolite examples that have clear links to cognitive dysfunction are spermidine (Eisenberg et al., 2009; Gupta et al., 2013), D-lactic acid (Mack, 2004; Sheedy et al., 2009; Munakata et al., 2010), and short-chained fatty acids (SCFAs- examples acetate, propionate-PPA, and butyrate-BA) (Macfabe, 2013). PPA and other elevated SCFAs are present in high amounts in the stool of ASD patients (Wang et al., 2010, 2012, 2014). Administration of PPA and BA causes ASD-like signs in animal models (Thomas et al., 2012; Foley et al., 2014a,b,c). The bacterial metabolite, 4-ethylphenylsulfate (4-EPS), is also abundant in the stool of ASD children (Persico and Napolioni, 2013), and likewise, can lead to ASD-like signs in mice treated with this metabolite (Hsiao et al., 2013). Ammonia, which is derived from cleavage of urea by bacterial ureases, is metabolized further by urea cycle in the liver. However, patients with liver disease can have excessive accumulation of ammonia leading to a condition called hepatic encephalopathy (Qureshi et al., 2014).

Many bacteria and their virulence factors can exploit and tamper with normal host neuroendocrine responses by suppressing them or even producing hormones or neurotransmitters that resemble those of the host. Examples of such include gamma-amino butyrate (GABA), norepinephrine (NE), serotonin, and dopamine (al Mardini et al., 1991; Li and Cao, 2010; Barrett et al., 2012; Cryan and Dinan, 2012). Host-produced neurochemicals, including NE and adrenaline, can speed up the rate of bacterial growth (Lyte, 2004, 2014; Karavolos et al., 2011). Instead of benefiting the host, host-produced L-DOPA can be usurped by *Helicobacteri pylori* to increase its rate of growth (Lyte, 2010). Antibiotic removal of this bacterium increases the amount of L-DOPA available for host neurocognitive responses.

The host hypothalamic-pituitary gland-adrenal (HPA) axis is vulnerable to gut microbiome alterations (Sudo et al., 2004). GF mice have increased circulating concentrations of ACTH and corticosterone, which can be mitigated by early but not later exposure to stool from SPF mice. 5-hydroxytryptamine and its primary metabolite 5-hydroxyindoleacetic acid are also present in high amounts in the hippocampus of GF animals (Clarke et al., 2013). GF rats exhibit behavioral abnormalities and elevated hypothalamic mRNA expression of *Crf* but decreased *Gr* mRNA in the hippocampus (Crumeyrolle-Arias et al., 2014). Dopaminergic turnover rate is also reduced in the frontal cortex, hippocampus, and striatum in these rats. Administration of the probiotic, *L. rhamosus* (JB-1), to mice decreased anxiety- and desperation- like behaviors, reduced corticosterone-induced stress response, and altered expression pattern of *Gaba*_*a*_*r* and *Gaba*_*b*_*r* in several brain regions (Bravo et al., 2011). The virulence factor, lipopolysaccharide (LPS), produced by *S. typhi* can activate host HPA axis, noradrenergic, and indoleaminergic systems (Dunn et al., 2003). In wild red squirrels (*Tamiasciurus hudsonicus*), high levels of fecal glucocorticoid metabolites were associated with lower bacterial diversity in the oral microbiome (Stothart et al., 2016). Administration of *Bifidobacterium pseudocatenulatum* CECT 7765 to C57Bl/6J previously subjected to maternal separation-induced stress modulates intestinal neurotransmitter and cytokine network to result in an improved HPA axis response (Moya-Perez et al., 2017).

The gut microbiome might induce various epigenetic changes in the brain, resulting in behavioral disruptions (Mischke and Plosch, 2013; Kumar et al., 2014; Stilling et al., 2014).

Bacterial-derived SCFAs, such as BA, PPA, and acetic acid can modulate epigenetic responses with BA the most potent SCFA inhibitor of histone deacetylases-HDAC (Candido et al., 1978; Davie, 2003). This class of enzymes removes acetyl groups from histone proteins, upon which these proteins are free to bind to DNA and compete with transcriptional factors. PPA, lactate, and pyruvate, are weak HDAC antagonists (Thangaraju et al., 2006; Waldecker et al., 2008; Latham et al., 2012). Conversely, the histone acetyl transferase (HAT) substrate availability was increased by acetate (Stilling et al., 2014). Supplementation of GF mice with SCFAs induced global histone acetylation and methylation and transcriptional responses in multiple host tissues (Krautkramer et al., 2016).

Beneficial gut microbes produce many of the B-vitamins required for normal host function, especially folate and vitamin B12. These micronutrients serve as methyl donors or factors in the methyl-cycle, which results in methylation of DNA and histone proteins (Leblanc et al., 2013). In pregnant women, the gut microbiota profile, especially relative amounts of Firmicutes and Bacteroidetes, are associated with leukocyte DNA methylation patterns of genes involved in lipid metabolism and obesity (Kumar et al., 2014). GF mice exhibited disruptions in postnatal 3′ CpG islands methylation patterns and gene activation and intestinal epithelial cells (Yu et al., 2015).

Gut bacteria might also govern other host epigenetic changes, for instance chromatin rearrangements and accessibility and disruptions in the expression of non-coding RNAs and RNA splicing factors (Bierne et al., 2012; Semenkovich et al., 2016). Gut microbiota may suppress host RNA polymerase II, an enzyme required for synthesis of coding and non-coding RNAs (Lutay et al., 2013). Select endosymbiotic bacteria appear to produce small non-coding RNAs that might affect host processes (Mayoral et al., 2014).

## Conclusions

The gut microbiome composition fluctuates throughout an individual's lifespan. Consequently, host condition and environmental state can guide which microbes predominate. Disruptions in the gut microbiome can in turn induce dramatic effects on host physiological responses and overall health. Thus, going forward environmental health scientists need to consider this key triangle or interactions between environmental chemical exposures, host, and gut microbiome. By inducing gut dysbiosis, such exposures can result in systemic and longstanding effects in the host.

Exposure to heavy metals, air pollutants, nanoparticles, and EDCs can lead to gut microbiome changes in various host taxa (Table 1). The data to date do not provide though any clear patterns as to whether certain bacteria are especially vulnerable to a range of chemical exposures. It is likely that the specific compound, dose, when the exposure occurs in the lifespan of the host, and duration of exposure elicit different microbiome effects. The ability of the gut microbiome to recover after removal of the chemical insult is uncertain. Ostensibly, exposure during the perinatal period, when microbes begin colonize the gut, is likely to induce more permanent effects than a similar exposure experienced at adulthood.

Exposure to some chemicals can mimic gut microbiome changes observed with other host environmental perturbations. For instance, BPA-exposure and consumption of a HFD led to similar bacterial disturbances in mice subjected to either treatment (Lai et al., 2016). How the gut microbiota fare in the face of multiple environmental challenges is uncertain. Only a few of the current studies have examined the effects of exposure to two or more chemicals. In ICR mice, combined exposure to arsenic and iron increased *Lactobacillus* spp., but this bacterial species decreased in mice exposed to either chemical alone (Guo et al., 2014). Additionally, combined exposure to both heavy metals eliminated changes in Brunesiella and Bacteroides that were identified with single exposure. Mongolian toads living in heavy metal contaminated water show several bacterial alterations compared to counterparts residing in a comparable unpolluted area (Zhang W. et al., 2016). Comparably, oral gavage of WT and Il10^−/−^ mice with a mixture of air pollutants from Ottawa resulted in host and gut microbiota alterations (Kish et al., 2013). Sprague-Dawley rats treated with DEP, MPB, TCS, or the combination of all three chemicals show similar gut microbiome changes at adolescence but such alterations are diminished by adulthood (Hu et al., 2016). Clearly, more work is needed to assess how host contact to multiple environmental chemicals, which better recapitulates real world scenarios, affects the gut microbiome in diverse taxa.

To facilitate a better understanding of how environmental chemicals affect the gut microbiome across a variety of species, a universal bioinformatics pipeline to analyze metagenomic data originating from different platforms and data repository site would be invaluable. Investigators could use such a database to compare gut microbe changes identified with their test environmental chemical(s) to previous studies, and thereby it would help determine whether certain gut microbes are more susceptible to certain toxicants regardless of their invertebrate or vertebrate host species. The Human Microbiome Project, which has been a useful tool in understanding how gut microbiome changes effect health and disease in humans (Human Microbiome Project Consortium, 2012a,b), could serve as a template for creation of such a database devoted specifically to gut microbiome changes induced by environmental chemical exposures in humans and animal model studies.

One of the other limitations of the current studies is that the gut microbiome changes are examined after exposure to one or two environmental chemicals. However, it is clear that there can be potential synergistic and antagonistic effects between environmental toxicants. Future studies should thus model as best as possible how the sum total of environmental toxicant exposure with those from different categories, otherwise considered the exposome paradigm (Dennis et al., 2016; Niedzwiecki and Miller, 2017; Sarigiannis, 2017), affects the gut microbiome in humans and animal models.

Another area that needs to be better understood is the contribution of environmental chemical-induced gut microbiome changes on altering phenotypic responses in the host, as illustrated in Figure 1. Correlation analyses may be useful in addressing this area. However, only two studies to date have attempted to parse out the involvement of such chemical-induced gut microbiome changes on downstream host effects. In non-agouti (*a/a*) mice offspring, intestinal flora changes were associated with an increase in body weight in males but not females (Wu et al., 2016). We showed that AgNP-induced changes in the gut microbiome were correlated with behavioral responses when the same male rats were tested in EPM (Javurek et al., 2017).

There might also be three-way interactions between environmental chemical exposure, host genotype and phenotypic responses, and gut microbiome, as depicted in Figure 1. Examples of how host responses can influence the gut microbiome are included in the Introduction. It is beyond the scope of this review to discuss how environmental chemicals might influence host responses. Host genetic, epigenetic, and phenotypic status and/or resident gut microbes might influence the pharmokinetics, including absorption, distribution, metabolism, and excretion of environmental chemicals. Gut bacteria are adapted to metabolize a variety of environmental chemicals, which can be achieved through azoreductases, nitroreductases, β-glucoronidases, sulfatases, and β-lyases. In so doing, microorganisms might reduce potential chemical toxicity to the host (Claus et al., 2016). While additional work is needed to determine how the gut flora affects host susceptibility to heavy metals, nanoparticles, and endocrine disruptors, there is strong evidence that this microbiome can influence the metabolism of plant phytoestrogens. Equol converting bacteria in the intestine, namely *Bacteroides uniformis*, modulate the chemopreventative properties of genistein (Atkinson et al., 2004, 2005, 2012; Lampe, 2009; Setchell and Clerici, 2010; Jackson et al., 2011; Renouf and Hendrich, 2011; Akaza, 2012; Macdonald and Wagner, 2012).

Finally, a better understanding of how environmental chemical-induced gut microbiome changes underpin host disease is essential. Disorders linked to gut dysbiosis include neurobehavioral, immunological, metabolic, gastrointestinal, cardiovascular, and likely many other disease states (Cenit et al., 2017; Quigley, 2017; Rieder et al., 2017; Shukla et al., 2017; Singh et al., 2017; Smolinska et al., 2017; Tang et al., 2017; Van De Wouw and Schellekens, 2017). In the case of brain, gut microbiome changes can lead to disturbances in this organ directly due to pathogenic spread of bacteria/virulence factors, bacterial metabolites, neuroendocrine disruptions, and/or epigenetic changes. An understanding of these pathogenic mechanisms is essential in prevention and remediation strategies. The current work has laid the groundwork to suggest a paradigm shift in that environmental chemical studies need to assess for pathological effects on the host and resident gut and other microbiomes that are all concurrently exposed and vulnerable to environmental chemical insults.

## Author contributions

CR wrote the manuscript and approved the final version.

### Conflict of interest statement

The author declares that the research was conducted in the absence of any commercial or financial relationships that could be construed as a potential conflict of interest.
